# Diagnosing congenital malaria in a high-transmission setting: clinical relevance and usefulness of *P. falciparum* HRP2-based testing

**DOI:** 10.1038/s41598-017-02173-6

**Published:** 2017-05-18

**Authors:** Hamtandi Magloire Natama, Delwendé Florence Ouedraogo, Hermann Sorgho, Eduard Rovira-Vallbona, Elisa Serra-Casas, M. Athanase Somé, Maminata Coulibaly-Traoré, Petra F. Mens, Luc Kestens, Halidou Tinto, Anna Rosanas-Urgell

**Affiliations:** 1 Department of Biomedical Sciences, Institute of Tropical Medicine, Antwerp, B 2000 Belgium; 2 Unité de Recherche Clinique de Nanoro, Institut de Recherche en Sciences de la Santé, Nanoro, BP 218 Burkina Faso; 3 Department of Biomedical Sciences, University of Antwerp, Antwerp, B 2610 Belgium; 4 Department of Medical Microbiology - Parasitology Unit, Academic Medical Centre, Amsterdam, 1105 AZ The Netherlands; 5 Centre Muraz, Bobo Dioulasso, BP 390 Burkina Faso

## Abstract

Congenital malaria diagnosis is challenging due to frequently observed low parasite density infections, while their clinical relevance during early infancy is not well characterized. In Nanoro health district (Burkina Faso), we determined the prevalence of congenital malaria by real-time quantitative PCR and we assessed the performance of rapid diagnosis test (RDT) and light microscopy (LM) to detect *Plasmodium falciparum* infections in cord-blood samples. In addition, we examined the usefulness of *P. falciparum* Histidine Rich Protein2 (*Pf*HRP2) as surrogate biomarker of infection and explored association between congenital malaria and clinical outcomes. A prevalence of congenital malaria by qPCR of 4% (16/400) was found, which increased to 10% among newborns from mothers infected at delivery. RDT and LM showed poor performances indicating limited utility for congenital malaria screening in cord blood. Because *Pf*HRP2 detection in cord blood could be affected by transplacental passage of parasite antigens, *Pf*HRP2 might not be used as a surrogate biomarker of congenital malaria infections. There was no evidence of a significant clinical impact of congenital malaria on infant’s health from birth to 59 days of life. Case control studies including long-term follow up may provide additional understanding on the relevance of neonatal malaria infections.

## Introduction

Congenital malaria is defined as the presence of *Plasmodium* asexual stages in newborn’s cord or peripheral blood during the first week of life as a result of materno-fetal transfer of malaria parasites^1^. The reported prevalence of congenital malaria in sub-Saharan Africa ranges from 0% to 54.2% depending on geographic area, endemicity, study design and detection method (i.e. light microscopy -LM- or Polymerase Chain Reaction -PCR-)^2–5^.

In malaria-endemic areas, symptomatic neonatal malaria infections are rare, suggesting spontaneous clearance of parasites detected at birth^1, 6–8^. However, some newborns with congenital malaria can develop symptoms within days^9–14^, weeks or months after birth^15–17^. Even though consequences of congenital malaria may be life-threatening little is known about the impact of these infections on the infant’s health, especially during the neonatal period (from birth to 28 days)^2, 13, 18, 19^. Targeted diagnosis of high risk newborns e.g. those born from infected mothers, could inform neonatal clinical care and guide prompt malaria treatment in clinically relevant cases.

In Burkina Faso, three studies have investigated the prevalence of congenital malaria by LM: two independent cross-sectional studies reported prevalences of 1.4–2.8% in cord blood samples^20, 21^, while a third study performed on hospitalized neonates during the first week of life reported a prevalence of 24.4%^9^. Routine diagnostic of clinical malaria in the country includes parasitological confirmation with rapid diagnostic test (RDT) and LM. Malaria parasite identification on Giemsa-stained blood smears is performed in district referral hospitals whereas the RDTs are the only diagnostic tool available for malaria at peripheral health centers. Although malaria RDTs have been implemented country-wide by the National Malaria Control Program (NMCP) since 2009, its performance for the detection of congenital malaria among neonates has not yet been evaluated.

In the present study, we determined the prevalence of congenital malaria, defined as cord-blood malaria infections as detected by quantitative PCR (qPCR) and we assessed the performance of a HRP2 RDT in plasma and LM to detect *Plasmodium falciparum* infections in cord blood samples. In addition, we used an Enzyme-linked Immunosorbent Assay (ELISA) kit to examine whether histidine-rich protein II antigen (*Pf*HRP2) levels are good surrogate biomarkers of congenital malaria infections. Finally, we explored the association between cord-blood malaria infections as detected by qPCR and clinical outcomes.

## Results

### Characteristics of study participants

Characteristics of study participants are presented in Table 1. In total, 400 mother-child pairs were included in the study. The mean age of the pregnant women was 26.2 ± 6.1 years and the majority were multigravida (gravidity ≥ 3). Approximately one third of all pregnant women had a peripheral infection at the time of delivery and another third had placental infection as detected by qPCR. Few cases of pre-term deliveries were recorded (2%). The majority of deliveries (93.5%) occurred during the rainy season (July to October) and the post-rainy season (November to February) corresponding to the highest and moderate transmission intensity periods in the country, compared with the dry season (March to June). Among newborns, there was a slightly higher but non-significant proportion of females than males (*P* = 0.317). Their overall mean birth weight was 3013.8 g and 11.3% of newborns had a low birth weight (<2500 g).

**Table 1:** Characteristics of the mother-child pairs^*^.

Characteristics	Value
Mothers age—years, Mean ± SD	26.2 ± 6.1
Mother’s gravidity—no. (%)	
Primigravidae	68 (17.0)
Secundigravidae	74 (18.5)
Multigravidae	258 (64.5)
Maternal peripheral infections at delivery (N = 390)—no. (%)	128 (32.8)
Maternal peripheral parasite density —Parasite/μL, Median (IQR)	22.7 (2.1–615.7)
Placental infections (N = 396)—no. (%)	137 (34.6)
Parasite density of placental infections—Parasite/μL, Median (IQR)	8.6 (1.2–388.5)
Matched peripheral and placental infection (N = 386)—no. (%)	
Peripheral positive/Placenta positive	99 (25.7)
Peripheral positive/Placenta negative	29 (7.5)
Peripheral negative/Placenta positive	34 (8.8)
Peripheral negative/Placenta negative	224 (58.0)
Gestational age at delivery—no. (%)	
Pre-term delivery (28–36 weeks)	8 (2)
Term delivery (>36 weeks)	392 (98)
Delivery period—no. (%)	
Rainy season	189 (47.25)
Post-rainy season	185 (46.25)
Dry season	26 (6.5)
Babies gender—no. (%)	
Male	190 (47.5)
Female	210 (52.5)
Babies birth weight—g, Mean ± SD	3013.8 ± 459.4
Low birth weight (<2500) —no. (%)	45 (11.3)
Fever episodes from birth to 59 days —no. (%)	42 (10.5)

^*^The characteristics are described for the 400 mother-child pairs, unless indicated otherwise.

SD, Standard Deviation; IQR, Interquartile Range; g, gram.

### Prevalence of congenital malaria by qPCR

The prevalence of congenital malaria, as detected by qPCR on cord-blood samples, was 4% (16/400) (Table 2). These infections were significantly associated with both maternal peripheral infections (OR = 16, 95% CI: 3.6–71.4; *P* < 0.001) and placental-blood infections (OR = 29.4, 95% CI: 3.8–225.8; *P* < 0.001). The prevalence of congenital malaria increased to 10.9% (14/128) among newborns whose mothers had peripheral infections at delivery and to 10.2% (14/137) among those born from mothers with placental-blood infections. Additionally, we performed histological examination of placental tissue from those mothers with cord-blood parasitaemia (Table 3). From the 16 samples positive by qPCR all presented with placental infection: half of them had a past placental infection and the other half an active placental infection. Parasite densities in the corresponding cord blood samples were <50 parasites/µL and >50 parasites/µL in past and active placental infections respectively. There was no significant difference in the prevalence of congenital malaria by gravidity (*P* = 0.980), although we observed a trend of decreasing prevalence in newborns from primigravid to multigravid women. The overall parasite densities in cord-blood infections cases were low with a geometric mean of 5.5 parasites/µL (95% CI: 1.4–22).

**Table 2:** Prevalence of congenital malaria as detected by qPCR.

Mother-child pairs	Congenital malaria		
	Cases detected	Prevalence (95% CI)	*P*-value*
**Total mother-child pairs (n = 400)**	16	4 (2.4–6.5)	—
**Maternal peripheral infection**			
Positive (n = 128)	14	10.9 (6.1–17.7)	<0.001
Negative (n = 262)	2	0.8 (0.1–2.7)	
**Placental infection**			
Positive (n = 137)	14	10.2 (5.7–16.6)	<0.001
Negative (n = 259)	1	0.4 (0.0–2.1)	
**Mother-child pairs according to gravidity**			
Primigravidae (n = 68)	3	4.4 (0.9–12.4)	0.980
Secundigravidae (n = 74)	3	4.1 (0.8–11.4)	
Multigravidae (n = 258)	10	3.9 (1.9–7.0)	

^*^ *P* value determined by Fisher’s exact test.

CI, confidence interval.

**Table 3:** Row data showing positive cord blood samples as detected by qPCR, RDT and/or ELISA (n = 20).

Subject categorization based on qPCR	Subject ID	Cord blood						Maternal blood		
		LM results	RDT results	ELISA results	ELISA mOD	ELISA Ag index	qPCR PD	Peripheral qPCR PD	Placental qPCR PD	Placental histology
qPCR positive	S44	Neg	Neg	Neg	0.048	0.2	150.4	143.8	22.4	Past infection
	S60	Neg	Neg	Neg	0.070	0.3	2.3	22.1	1.2	Past infection
	S78	Neg	Neg	Neg	0.130	0.5	26.1	7.0	48.2	Past infection
	S89	Neg	Neg	Neg	0.164	0.7	0.2	10150.8	9275.2	Active infection
	S97	Neg	Neg	Neg	0.038	0.2	1.0	0.0	0.0	Past infection
	S102	Neg	Neg	Neg	0.042	0.17	0.6	1178.0	39.0	Past infection
	S107	Neg	Neg	Neg	0.063	0.2	0.3	256.2	2909.1	Active infection
	S116	Neg	Neg	Neg	0.039	0.2	309.7	0.0	NA	Active infection
	S127	Neg	Neg	Neg	0.070	0.3	0.3	15.0	1.0	Past infection
	S129	Neg	Neg	Neg	0.051	0.21	340.5	111.0	248.5	Active infection
	S151	Neg	**Pos**	**Pos**	3.280	13.45	5.3	1593.6	673.0	Active infection
	S170	Neg	Neg	Neg	0.045	0.16	0.7	16.3	0.5	Past infection
	S173	Neg	Neg	Neg	0.043	0.18	34.0	733.7	456.0	Active infection
	S201	Neg	**Pos**	**Pos**	3.240	13.32	79.8	40.4	93.2	Active infection
	S229	Neg	Neg	Neg	0.137	0.55	2.2	625.3	550.1	Active infection
	S251	Neg	Neg	Neg	0.052	0.21	4.4	2.2	11.0	Past infection
qPCR negative	S33	Neg	**Pos**	**Pos**	3.930	16.6	Neg	1489.2	14341.8	Active infection
	S91	Neg	Neg	**Pos**	0.316	1.3	Neg	8626.0	13218.4	Active infection
	S100	Neg	Neg	**Pos**	0.350	1.4	Neg	17998.7	15400.0	Active infection
	S112	Neg	Neg	**Pos**	0.302	1.2	Neg	32633.0	5448.4	Active infection

The data includes the mean optical density and the derived antigen index by ELISA, placental histology results and, parasite densities in cord, maternal peripheral and placental blood. qPCR, quantitative real-time Polymerase Chain Reaction; LM, Light Microscopy; RDT, rapid diagnostic test; ELISA, Enzyme-Linked ImmunoSorbent Assay; mOD, mean optical density; Ag index, Antigen index obtained by dividing the mOD of each sample by the cut-off value; PD, parasite density (number of parasites/µL) as calculated by qPCR; ID, identification; NA, Not available; Pos, positive (in bold); Neg, negative.

### Diagnostic accuracy of RDT and LM for congenital malaria diagnosis

HRP2 was detected in cord-blood in 3 (0.75%) out of the 400 plasma samples tested by RDT, while LM failed to detect any infection in the study population (Table 3). Overall, RDT and LM accuracy to detect cord-blood infections was poor as compared to qPCR as gold standard (Table 4). RDT missed 87.5% (14/16) of cord blood infections resulting in a sensitivity and specificity values of 12.5% and 99.7% respectively. Among infants who had a positive RDT positive predictive value (PPV), *i.e*. the probability of having a qPCR-confirmed congenital malaria infection, was 66.7% (95% CI: 12.5–98.2%) while negative predictive value (NPV), *i.e*. the likelihood of not having the infection among those who had a negative RDT, was 96.5% (95% CI: 94–98%). There was a limited agreement between RDT and qPCR with a Cohen’s kappa coefficient equal 0.2.

**Table 4:** Congenital malaria detection in cord blood samples according to LM, RDT and ELISA compared to qPCR.

Diagnostic tools	Total	qPCR		Sensitivity (95% CI)	Specificity (95% CI)
		Positive (n = 16)	Negative (n = 384)		
**RDT**					
Positive	3	2	1	12.5 (2.2–39.6)	99.7 (98.3–100)
Negative	397	14	383		
**LM**					
Positive	0	0	0	0 (0–24.1)	100 (98.8–100)
Negative	400	16	384		
**ELISA**					
Positive	6	2	4	—	—
Negative	394	14	380		

qPCR, quantitative real-time Polymerase Chain Reaction; LM, Light Microscopy; RDT, Rapid Diagnostic Test; ELISA, Enzyme-Linked ImmunoSorbent Assay; CI, Confidence Interval.

### *Pf*HRP2-levels in cord blood: antigen based testing and association with parasite densities

ELISA test for *Pf*HRP2 antigen was positive in 6 (1.5%) out of the 400 tested cord blood samples (Table 3), although only 2/6 presented a confirmed qPCR cord-blood infection (Table 4). Among the 6 ELISA-positive samples, 3 were also positive by RDT performed in stored cord plasma samples (Table 3). RDT and ELISA were both positive when the antigen index determined by the ELISA test was >13 (Table 3). Indeed, samples that were found positive by RDT had a significantly higher antigen index than samples only found positive by qPCR and/or ELISA (14.5 versus 0.5, *P* = 0.007).

**Figure 1:**
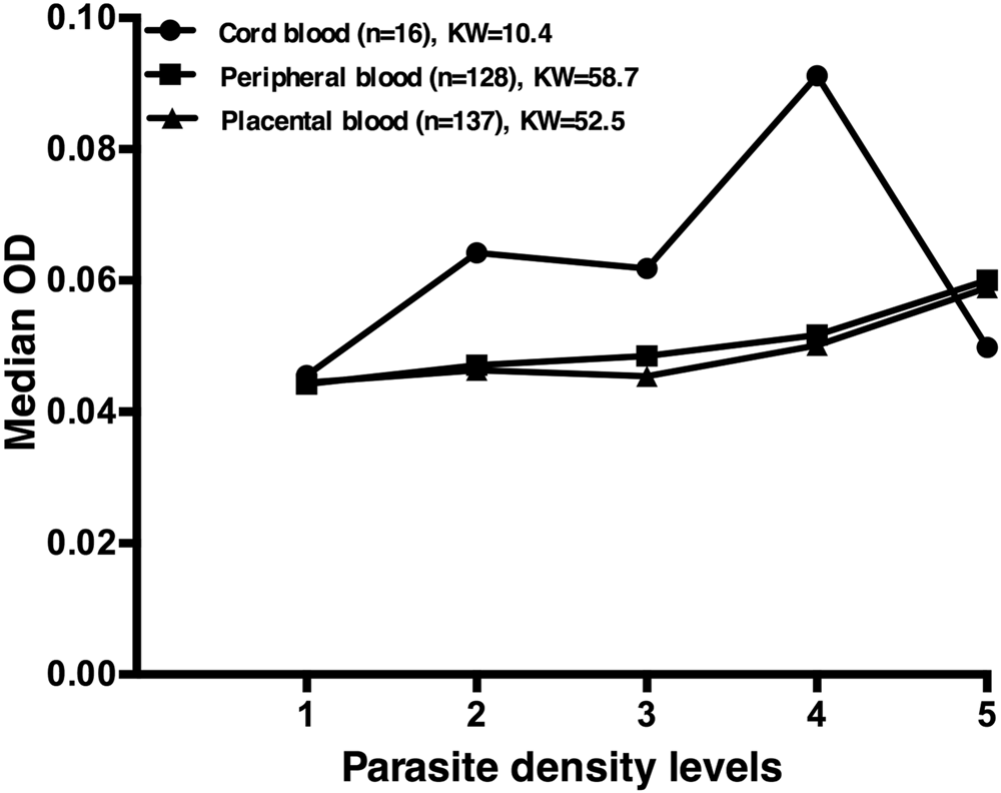
*Pf*HRP2 levels and parasite density: variation of the median optical density (OD) for *Pf*HRP2 detection by ELISA in cord blood according to parasite density levels in cord, maternal peripheral and placental blood by qPCR. Parasite densities were categorized using interquartile ranges (IQR) as follows: 1 = negative; 2 = 0 to IQR1; 3 = IQR1 to IQR2; 4 = IQR2 to IQR3 and 5 = IQR3 to the maximum; KW, Kruskal Wallis coefficient.

In order to assess the appropriateness of using *Pf*HRP2 as a biomarker for detection of congenital *P. falciparum* malaria infection, we investigated whether neonatal *Pf*HRP2 levels in plasma were associated with the parasite densities in cord blood. The same association was tested for maternal blood parasite densities. The results suggested that the optical density (OD) of the *Pf*HRP2-ELISA test could be associated with parasite densities from both cord and maternal blood, although OD values were much strongly associated with parasite densities in the mother (Kruskal Wallis coefficients = 52.5 and 58.7 for peripheral and placental blood respectively; *P* < 0.001) than those in cord blood (Kruskal Wallis coefficient = 10.4; *P* = 0.034). Indeed, as shown in Fig. 1, median *Pf*HRP2 ODs followed a consistent trend towards increasing parasite densities in the placenta and maternal peripheral blood, while cord antigen levels showed a markedly different profile when compared to parasite density levels in cord blood. The strong correlation between cord antigen detection and the maternal parasite burden was further confirmed by comparing maternal parasite densities among ELISA-positive and qPCR-positive cases. As shown in Table 5, parasite densities, both in the placenta and in the peripheral blood, were significantly higher in mothers with a positive cord blood sample ELISA test (*P* = 0.001) than those with congenital malaria infection detected by qPCR (*P* < 0.05).

**Table 5:** Parasite density in maternal peripheral and placental blood among congenital malaria cases detected.

Cord blood tests	N	Placental infection			Peripheral infection	
		Parasite density* Median (IQR)	P**	N	Parasite density* Median (IQR)	P**
**qPCR**						
Positive	14	70.7 (10.8–550.1)	0.118	14	127.4 (16.3–733.7)	0.114
Negative	123	6.8 (1.2–388)		114	20.8 (1.6–596)	
**ELISA**						
Positive	6	9333.4 (673–14341.8)	0.001	6	5109.8 (1489.2–17998.7)	0.001
Negative	131	6.8 (1.2–343.5)		122	20.8 (2.1–561.3)	

^*^Parasite density (number of parasite/μL) determined by qPCR.

^**^ *P* value determined by the Mann-Whitney test.

qPCR, quantitative real-time Polymerase Chain Reaction; ELISA, Enzyme-Linked ImmunoSorbent Assay; IQR, Interquartile Range.

### Congenital malaria by qPCR and clinical outcomes

We investigated whether congenital malaria infections as detected by qPCR in cord-blood samples were associated with low birth weight (LBW), occurrence of fever and risk of malaria during the first two months of life. The overall prevalence of LBW in the study population was 11.3% (45/400 newborns). Although 18.7% (3/16) of the newborns with cord-blood infections were born with LBW, the association was non-significant (OR = 1.9, 95% CI: 0.5–6.9; *P* = 0.264). One neonate with congenital malaria by qPCR, developed clinical malaria at day 36 of the follow-up. *Msp1* and *msp2* genotyping of the paired samples, *i.e*. at birth and day 36, confirmed a new infection at day 36 indicating that the cord-blood infection at birth was cleared spontaneously without developing into a clinical case. In total, 10.5% (42/400) of children experienced at least one fever episode during the follow-up period and only two of them had cord-blood malaria infection. Finally, there was no evidence of association between fever episodes during the first two months of life and cord-blood infections (OR = 0.6, 95% CI = 0.3–5.4; *P* = 0.530).

## Discussion

In this study we found a 4% prevalence of congenital malaria as detected by qPCR in cord-blood samples from mothers delivering in the Nanoro health district of Burkina Faso. Previous studies from Burkina Faso using LM to detect congenital malaria infections have reported lower prevalence estimates of 2.8% in Ouagadougou^21^ and 1.4% in Boussé & Koupéla^20^, while a 24.4% prevalence was reported in a 10-year retrospective study, including only hospitalized sick neonates (<7 days)^9^. Several studies have discussed the impact of seasonality, diagnostic method and study design on the estimated prevalence of congenital malaria^3^. Although our study recruitment period is comparable to previous reports in the country^20, 21^, the qPCR technique we used, which targets *Pfvar* genes (≈60 copies/genome)^22^, is 10 times more sensitive than the standard 18S rRNA-based qPCR used in other studies^23, 24^ and could explain at least partially variability observed between studies. On the other hand, the current study is ancillary to a larger trial investigating the effectiveness of strategies for malaria prevention in pregnancy^25^. Uptake of pregnancy preventive and control measures such as intermittent preventive treatment of malaria in pregnancy with sulfadoxine-pyrimethamine (IPTp-SP), insecticide treated net, malaria diagnosis and treatment is expected to be higher in the study population than in the general population due to the trial effect. These preventive measures, which are known to be effective in preventing maternal and placental malaria infections^26–30^, may have reduced the risk of infection and transplacental transmission of malaria parasites potentially leading to underestimation of congenital malaria prevalence.

Indeed, cord-blood infections as detected by PCR were more often detected among mothers with maternal peripheral and placental infections at delivery (10.9% and 10.2% respectively) than in the general study population (4%). Although we cannot exclude that a number of these cord blood infections are due to admixture with maternal or placental blood at delivery, evidences of a compromised placenta, which increases the risk of transplacental passage^31–33^, found in all detected cord-blood infections and discordant levels of malaria parasitemia between cord and placental/maternal blood (with higher levels in cord than placental/maternal blood) are in agreement with malaria infections acquired antenatally^24^.

Therefore, due to the increased risk of congenital malaria found among newborns from infected women at delivery, this high risk group could be targeted for congenital malaria screening to allow for prompt and adequate neonatal clinical care. In this scenario, malaria diagnosis using umbilical cord-blood sample would be the best option to avoid invasive blood sample collection of the newborn. However, detection of congenital malaria infections in cord blood samples using field implemented diagnostic tools such RDT and LM remains a challenge.

LM is the standard method for malaria diagnosis in Burkina Faso and is available at the health district referral hospitals. In this study, LM did not detect any umbilical cord blood infections, contrasting with previous reports from Burkina Faso^20, 21^. The observed differences can be explained by the lower cord blood parasite densities detected by qPCR in our study (5.5 [95% CI, 1.4–22] parasites/µL) compared with parasite densities detected by LM in the other studies (315.69 [95% CI, 87.02–1,145.20] parasites/µL and 1444 [range: 231–5,102] parasites/µL, respectively^20, 21^). Lower parasite densities could also be explained by a higher uptake of pregnancy preventive measures in the present study. Thus, LM does not appear as an optimal tool to screen for congenital malaria infections in cord blood samples. Indeed, limited sensitivity of LM to detect congenital malaria infections compared to molecular methods has also been observed in studies conducted in other African countries^7, 23, 34^.

The SD-Bioline *Pf*HRP2 is the RDT recommended by the NMCP in Burkina Faso for malaria diagnosis in peripheral health centers in rural areas where there are no laboratory facilities available. Here, we assessed whether the use of *Pf*HRP2-RDTs could be extended to screen umbilical cord-blood samples for *P. falciparum* detection. SD-Bioline *Pf*HRP2 RDT missed 87.5% of umbilical cord-blood infections detected by qPCR indicating that diagnostic performance to detect congenital malaria is suboptimal. Moreover, RDT test was only found to be positive when *Pf*HRP2 levels were very high (RDT positives >13 vs qPCR/ELISA positives <2). Of note, we did not follow manufacturers’ instructions in the use of the *Pf*HRP2 RDT as we tested stored cord plasma samples instead of whole blood, which may have decreased test sensitivity. However, we found a good agreement between RDT and ELISA tests taking into account that RDT uses lower plasma volumes than ELISA. This is in agreement with previous reports^35^ and indicates that results obtained here are reliable. In the other hand, although we cannot exclude that RDT- or ELISA-negative/PCR-positive samples are due to the presence of *pfhrp2*-deleted *P. falciparum* parasites^36^, parasite densities detected by qPCR in cord blood samples are low and often under the limit of detection of the used RDT. Nevertheless, future studies should investigate the prevalence of *pfhrp2*-deletion particularly in areas of high malaria transmission as in the study area.

Only two other studies have previously evaluated RDT performance to detect congenital malaria infections^37, 38^. In Nigeria, pLDH-Optimal® RDT did not detect any congenital malaria infection while prevalence by LM was 10.9%^37^ and, in Burundi two newborns were found positive by SD-Bioline *Pf*HRP2 but none by CareStart pan-pLDH^38^. When samples from these two newborns were subsequently tested by qPCR, both resulted negative and were resolved as false RDT-positives, even though WHO RDT performance evaluation reported no false positive SD Bioline Pf-antigen results after testing 140 true-negative samples^39^.Since *Pf*HRP2-based tests sensitivity depends on antigen concentration and this seems to be determined by the parasite burden^40, 41^, we expected positive RDT cases among samples with high parasite density in umbilical cord blood. Surprisingly, congenital malaria infections presenting higher parasitemia (*i.e*. >100 parasites/µL; n = 3) were all negative by RDT, while RDT-positive cord-blood samples presented 79.8, 5.3 and 0 parasites/µL. Of note, the RDT-positive/qPCR-negative cord sample corresponded to a mother with very high parasite burden at the time of delivery (14,342 parasites/µL and 1,489 parasites/µL in their placental and peripheral blood, respectively). This observation is in line with the findings reported in the study from Burundi^38^, where the two false-positive RDT results also corresponded to newborns from mothers with very high peripheral parasitemia (*i.e*. 99,520 and 454,061 parasites/µL). Indeed, a significant correlation between cord *Pf*HRP2 levels and maternal parasite burden was further confirmed by our results. On the one hand, median placental and peripheral densities were found to be significantly higher among mother-child pairs with ELISA-positive result (*P* = 0.001) but not among those with a cord qPCR-positive result. In addition, cord *Pf*HRP2 levels correlated strongly with maternal parasite densities than cord blood parasite densities. Unfortunately, we had no data available to investigate the direct correlation between cord blood and maternal *Pf*HRP2 levels at delivery. All these evidences suggest that *Pf*HRP2 in cord blood may be the result of the transplacental passage of maternal antigens rather than a reflection of concurrent malaria infection in the newborn. Hence, detection of congenital malaria infection based on the presence of *Pf*HRP2 antigen might not be a reliable approach.

Transplacental passage of malaria antigens was previously reported in a study conducted in The Gambia that found GLURP antigen in 28.6% of tested cord sera samples^42^. Likewise, transplacental transfer of circulating antigens has been also documented in other parasitic diseases such as lymphatic filariasis^43, 44^. However, in the present study high maternal parasite densities do not always correlated with high *Pf*HRP2 levels and positive RDT and/or ELISA results suggesting that transplacental passage of parasite antigens can be influenced by different conditions during pregnancy. In fact, factors that contribute to mother-to-child transmission of malaria parasites (or antigens) have not yet been well elucidated, although pre-existing level of malaria immunity, history of maternal infections and malaria-related placental changes and damage seems to play an important role^31–33, 45–48^. In summary, our results indicate that *Pf*HRP2 levels in cord blood samples are not a good surrogate biomarker of *P. falciparum* infections. Hence, even if high-sensitive *Pf*HRP2-based RDTs are available on the market in the near future^49^, these would not render a useful tool for parasite presence detection in cord blood. Further investigations should include quantification of *P. falciparum* antigens levels such as (but not limited to) *Pf*HRP2 in maternal peripheral, placental and umbilical cord blood samples.

Although there was a trend towards lower birth weight among newborns with congenital malaria, we did not find a significant association between LBW and cord-blood infections as detected by qPCR. The lack of association could be related to the low number of LBW newborns observed in our study population. We did not observe any case of clinical congenital malaria (presence of malaria parasites in symptomatic newborn during the first week of life) neither clinical neonatal malaria (parasites and symptoms from 1 to 4 weeks of life). One newborn with congenital malaria developed a clinical malaria infection 36 days after birth. However genotyping of paired samples concluded that the clinical episode at day 36 was due to a new infection.

Overall, the lack of a significant clinical impact of congenital malaria as detected by PCR on infant’s health during the first 2 months of life in our study, may be due to the low number of congenital malaria infections. In the other hand, these results are concordant with other studies in high malaria transmission areas of sub-Saharan Africa, where symptomatic neonatal infections are rare suggesting that congenital malaria is infrequently associated with clinical disease^6, 8, 18^. The importance of maternal derived antibodies in controlling infections early in infancy could be one of the main explanations for the clearance of infections and the lack of association with symptoms and adverse outcomes after birth^50^. In addition, as in all studies based on high-sensitive detection of parasite DNA or antigen, we are indirectly detecting parasite material rather than alive parasites. Therefore, we cannot exclude that some of the positive results correspond to non-viable parasites^51^.

Prenatal exposure to blood-stage malaria parasites/antigens may have profound long-term effects during infancy and childhood by priming the immune responses of the fetus and/or by inducing immune tolerance^24, 52^. However, the role of congenital malaria in the modulation of that immune response is unknown. Hence, a long-term effect of congenital malaria on newborns health living in high malaria endemic settings cannot be excluded and deserves further investigations. In the meantime, prenatal exposure to malaria parasites/antigens should be prevented through effective strategies for malaria control in pregnancy.

In conclusion, congenital malaria infection is not uncommon in Nanoro health district and the estimate of its prevalence using a high sensitive qPCR could reach 10% among newborns from mothers presenting peripheral and/or placental infections at delivery. The overall poor performance of SD-Bioline *Pf*HRP2 RDT compared to qPCR suggests a limited utility of RDTs for congenital malaria screening in cord-blood samples in Burkina Faso. In addition, because *Pf*HRP2 detection in cord blood could be influenced by high levels of parasite antigens in maternal blood through a transplacental passage, *Pf*HRP2 might not be used as a surrogate of congenital malaria infection. There was no evidence of a significant clinical impact of congenital malaria infections on infant’s health from birth to 59 days of life. Case-control studies including long-term follow up may provide additional understanding on the relevance of neonatal malaria infections.

## Methods

### Study site

This study was conducted in 10 peripheral health centers of Nanoro Health District (NHD) in Burkina Faso. The NHD comprises approximately 153,000 inhabitants and is located 85 km west of Ouagadougou, the country’s capital. Malaria transmission in the region is seasonal and hyperendemic with the highest transmission period overlapping with the rainy season from July to October. The mean prevalence of malaria in the country is estimated to 62.2% with a mean entomological inoculation rate of 118.1 per person per year^53^.

### Study participants

This study is ancillary to a larger multicentric trial assessing the effectiveness of malaria in pregnancy preventive strategies in Burkina Faso, Benin and The Gambia. Details of the main trial protocol have been described elsewhere^25^. For the present study, 400 mother-child pairs were enrolled at the moment of delivery (from July 2014 to April 2015) after inform consent from the mothers was obtained and newborns were followed-up by passive case detection for 59 days to identify occurrence of fever or clinical malaria cases. Sample size was estimated assuming a minimum congenital malaria prevalence of 1.4% as reported in a previous study in Burkina Faso^20^. Ethical approvals were obtained from institutional ethics committees at Centre Muraz, Bobo Dioulasso, Burkina Faso (006–2014/CE-CM), Institute of Tropical Medicine, Antwerp, Belgium (953/14) and University Hospital in Antwerp (UZA), Belgium (14/26/277). All procedures followed were carried out in accordance with the Helsinki Declaration as revised in 2013.

### Sample collection

At delivery, 200 µL of peripheral blood was obtained from the mother by finger-prick and placental blood was collected by pipette aspiration after incision of the maternal side of the placenta. A placental tissue section was collected from the maternal side and stored into 10% neutral buffer formalin. Cord blood was collected in heparin containing tubes by venipuncture of the umbilical vein and used to prepare thick blood smears for LM examination. Blood drops of all samples were spotted onto filter paper for posterior *P. falciparum* diagnosis by qPCR. One plasma aliquot was prepared from cord blood for ELISA and RDT test performance and stored at −80 °C. Newborns visiting the health center with presence or history of fever during the previous 24 hours were screened for malaria infection with RDT and, if positive, treated with artesunate-amodiaquine or artemether-lumefantrine according to national guidelines. In addition, a capillary blood sample was collected from finger-prick for qPCR analysis and LM examination.

### Malaria Microscopy

Thick blood films stained with Giemsa (10%) were used for malaria detection by LM according to standard procedures^54^. Parasite density was expressed as the number of asexual parasites per μL of blood based on an assumed 8000 white blood cells per μL of blood. A slide was considered negative if no parasites were seen after examining 100 fields. Each slide was read by two independent experienced microscopists blinded to each other’s results and in case of discrepancy, the slide was read by a third experienced microscopists. After the completion of the reads an internal quality control was performed by a fourth experienced reader for 10% of slides. Slides of the quality control included all positive samples detected by qPCR. Histological examination of placental tissue collected at delivery was performed as described elsewhere^33^.

### Rapid Diagnostic Test for malaria

RDT SD-Bioline malaria antigen P.f® test (Standard Diagnostics, Inc, Korea) detecting *Pf*HRP2 was used. Umbilical cord plasma was thawed at room temperature, centrifuged at 500 g for 10 min and five microliters were transferred into the RDT sample well, to which four drops of assay diluent were added. The RDT result was read in 15–30 min.

### Enzyme-linked ImmunoSorbent Assay (ELISA) for *Pf*HRP2

Detection of *Pf*HRP2 antigen was performed with Malaria Ag CELISA^TM^ kit (Cellabs, Brookvale, New South Wales, Australia) following manufacturer’s recommendations. Briefly, 100 µL of umbilical cord plasma were tested and optical density (OD) read at 450 nm with Multiskan^TM^ FC Microplate Photometer (Thermo Scientific). All samples and controls were tested in duplicate. Positive and negative controls were provided by the manufacturer and OD values were normalized using a reference positive control in all plates. Samples with a mean OD value above the cut-off level (OD of negative control +0.2 unit) were considered positive for *Pf*HRP2 antigen as recommended by the manufacturer. The antigen index values were determined by dividing the mean OD value of each sample by the cut-off value as previously described^55^.

### *varATS* real-time PCR (qPCR) for *P. falciparum* detection and quantification

For the molecular diagnosis by qPCR, filter papers with dried blood samples from maternal peripheral, placental and cord blood were punched and three circles of 5 mm in diameter was used for DNA extraction with QIAamp 96 DNA blood kit (Qiagen, Germany). Extracted DNA was eluted in 150μL of water. Five microliters of DNA were used for qPCR analysis targeting *P. falciparum* var gene acidic terminal sequence (varATS, ≈59 copies per genome) as previously described^22^. The limit of detection in our laboratory was 0.1 parasite/μL using DNA extracted from blood spot on filter paper. Parasite densities were obtained by extrapolating cycle thresholds (Ct) from a standard curve prepared with titrated samples containing known numbers of infected erythrocytes diluted in whole blood (100,000 to 0.1 parasites/μL). Samples with Ct value ≤39.7 were considered positive.

### Parasites genotyping

Nested PCR targeting *P. falciparum msp1* and *msp2* genes was used to genotype parasite clones in a paired-sample corresponding to cord blood and peripheral blood of a newborn that experienced a malaria infection during the study follow-up. Family specific primers for *msp1* (K1, MAD20 and R033) and *msp2* (FC27 and IC(3D7)) were used according to the genotyping procedures recommended by WHO^56^.

### Data analysis and definition of terms

Data were double entered into the study databases (OpenClinica, community version or Excel, Microsoft, USA) and analyzed using Epi Info version 3.5.4 and STATA 12.0 (StataCorp, USA). Normally distributed data was described by mean ± SD and non-normally distributed data was described by the median with interquartile range (IQR). Congenital malaria prevalences were calculated with 95% Confidence Interval (CI). Parasite densities and ELISA optical densities (ODs) were not normally distributed even after logarithm transformation. Thus, the difference in median values of parasite density and ODs between groups were analyzed using the non-parametric Mann-Whitney and Kruskal Wallis tests. Sensitivity and specificity were calculated with 95% CI to compare RDT and LM against qPCR. *P* values < 0.05 were considered statistically significant. The following definitions were applied: (a) Congenital malaria infection: cord-blood malaria infections as detected by quantitative PCR; (b) Maternal peripheral and placental infections: presence of parasite DNA detected by qPCR in the peripheral and placental blood at delivery; (c) Low birth weight: <2,500 g; (d) Fever: axillary temperature ≥37.5 °C; (e) Clinical malaria episode: presence of fever at the moment of screening or during the previous 24 h and positive test by RDT, LM or qPCR.
